# Semmelweis Caring University Model Program Based on the Development of a Center of Preventive Services: Health for All Employees at a University Occupational Setting

**DOI:** 10.3389/fpubh.2021.727668

**Published:** 2021-11-29

**Authors:** Zoltán Ungvári, Róza Ádány, Attila J. Szabó, Gabriella Dörnyei, Mariann Moizs, György Purebl, László Kalabay, Péter Varga, Péter Torzsa, Miklós Kellermayer, Béla Merkely

**Affiliations:** ^1^International Training Program in Geroscience/Healthy Aging Program, Department of Public Health, Semmelweis University, Budapest, Hungary; ^2^Vascular Cognitive Impairment and Neurodegeneration Program, Center for Geroscience and Healthy Brain Aging, Department of Biochemistry and Molecular Biology, University of Oklahoma Health Sciences Center, Oklahoma City, OK, United States; ^3^Department of Health Promotion Sciences, The Hudson College of Public Health, University of Oklahoma Health Sciences Center, Oklahoma City, OK, United States; ^4^MTA-DE Public Health Research Group, Department of Public Health and Epidemiology, Faculty of Medicine, University of Debrecen, Debrecen, Hungary; ^5^First Department of Pediatrics, Faculty of Medicine, Semmelweis University, Budapest, Hungary; ^6^MTA-SE Pediatrics and Nephrology Research Group, Semmelweis University, Budapest, Hungary; ^7^Department of Morphology and Physiology, Faculty of Health Sciences, Semmelweis University, Budapest, Hungary; ^8^Somogy County Móricz Kaposi Teaching Hospital, Kaposvár, Hungary; ^9^Institute of Behavioral Sciences, Faculty of Medicine, Semmelweis University, Budapest, Hungary; ^10^Department of Family Medicine, Faculty of Medicine, Semmelweis University, Budapest, Hungary; ^11^Clinical Center, Semmelweis University, Budapest, Hungary; ^12^Department of Biophysics and Radiation Biology, Faculty of Medicine, Semmelweis University, Budapest, Hungary; ^13^Heart and Vascular Center, Semmelweis University, Budapest, Hungary

**Keywords:** Semmelweis University, Caring University Model Program, employees, health risk assessment, occupational setting, multiprofessional public health team, counseling services, health promotion

## Abstract

The leadership of the Semmelweis University as a leading institution of higher education in Hungary and the Central Eastern European region within the area of medicine and health sciences has decided to reflect on the unfavorable public health situation in the country as well as the deteriorating health behavior and health status indicators in the Hungarian population by the development of an occupational setting-based personalized public health model program targeting its about 8500 employees. Based on its infrastructure and human resources the core element of the program is the establishment of the Center of Preventive Services (CPS) with units providing health risk assessment for each employee, and whenever necessary consultation with medical specialist in preventive medicine and public health, as well as counseling with dietician, physiotherapist and/or health psychologist. The service providers are the staff members of the relevant faculties in collaboration with partner primary and occupational care physicians. The units of the CPS can also serve as practical training sites for students at various levels of medical and health sciences training, and strongly contribute to the development and improvement of their skills to be able for working as a team in service provision. The employees are not only beneficiaries of health risk assessment and screening repeated on a regular basis and adequate interventions at the right time, but they also serve as a sample for a longitudinal cohort study and further *ad hoc* surveys for defining and implementing interventions to support health protection, disease prevention and healthy aging among them.

## Introduction

Health status of the Hungarian population is among the least favorable in the European Union. Life expectancy at birth exceeds 80 years in almost two-thirds of EU countries, but still remains at only around 76 years in Hungary, Serbia, Bulgaria, Latvia, and Romania (1). The life expectancy at birth in Hungary is as low as 76.5 years for 2019 in comparison with 81.3 years EU average (1, 2). The gap between the EU average and the Hungarian figures is 5.4 years for males (78.5 years vs. 73.1 years) and 4.3 years for females (84.0 years vs. 79.7 years). Regarding the causal structure of premature mortality the non-communicable diseases dominate; in 2016 the representation of malignant and cardiovascular diseases among the causes of early death was 34% and 33% for males, while 47% and 24% for females, respectively (3).

A number of risk factors, such as smoking (4), unhealthy nutrition (5) and physical inactivity (6) show very high prevalence in the Hungarian population, and consequently—as the OECD reported for 2016–Hungary has the highest obesity rate in Europe, and only the populations of the United States, Mexico and New-Zealand are more obese than Hungarians (7). The prevalence of metabolic syndrome (MetS), the most robust indicator of increased risk for cardiometabolic diseases was found as high as 39.8%, and that of insulin resistance characterized by HOMA-IR as 48.1% in the Hungarian population aged 20–64 years in a complex health (behavior and examination) survey in 2018 (8). It is important to mention that the trend of change in the prevalence of metabolic syndrome is very unfavorable: the prevalence of MetS increased significantly in the period between 2006 and 2018 (from 34.9% to 42.2%, *p* = 0.035) due to the increased prevalence of raised blood pressure (from 45.6% to 57.0%, *p* = 0.002) and raised fasting glucose concentration (13.2% vs. 24.8%, *p* < 0.001). It is even more unfavorable that the increase mainly affected the younger (20–34 years old) age group (12.1% in 2006 vs. 31.6% in 2018, *p* = 0.001). It is also worth mentioning that while the prevalence of MetS and its components has increased significantly, the prevalence of preventive medication is unchanged (antihypertensive and antidiabetic treatments) or even significantly decreased (lipid-lowering medication) indicating poor performance at the level of not only primary, but also secondary prevention (9).

The latest figures demonstrate the burden of malignant diseases is very severe in Hungary; mortality rate for both sexes from all cancers (averaged 149 deaths per 100,000) is the highest in Hungary among all countries worldwide, and it has first place in rankings of lung, colorectal and pancreas cancer deaths. The fact that although cancer morbidity is also very disadvantageous, the average incidence rate (338.2/100,000 population) is much lower than that is in some other countries with more favorable mortality figures (10), indicates that not only primary cancer prevention, but also cancer screening and/or treatment should be markedly improved.

Certainly, it cannot be assumed that the population of University employees is representative of the general Hungarian population, but it is reasonable to suppose that similar health and health behavior problems exist among them. The only one questionnaire-based *ad hoc* survey among employees of the Semmelweis University was carried out on a randomly selected sample (*n* = 1,085; average age 44.8 years; 27% males, 73% females) in 2019. Almost one third of the university employees (32%) perceived their health to be bad or very bad, and 44% of them mentioned to be affected by certain type(s) of chronic non-communicable diseases. The proportion of physically inactive people is estimated at 38% and only 17% of them engage in regular leisure time physical activity, while the prevalence of smoking is 16%. The average BMI value is 25.7 kg/m^2^ (females: 24.7 kg/m^2^; men 27.6 kg/m^2^) (unpublished data from Magor Papp MD, the head of the Health Promotion Centre).

As the latest country report of the European Commission describes Hungary had the third highest preventable mortality rate and the fifth highest amenable mortality rate in the EU in 2016, which clearly indicates substantial room for improvement through more effective preventive interventions and adequate timely health care (11). In spite of severe challenges, public health is not on the health policy agenda in Hungary; previously the turbulent political debate on the reform of the health care system detracted almost all attention and resources (12).

Increasing prevalence of cardiometabolic risk factors (8, 9), and the fact that Hungary is among the three countries most affected by the COVID 19 pandemic regarding the death rate for one million population (13) substantiates the concern that the health status of Hungarians will further be deteriorated in the post-COVID period.

It cannot be emphasized enough how important and urgent it is to intensify public health interventions. Among them, health promoting activities at population level as well as at different settings to protect and promote health and strongly reduce preventable and amenable mortality caused by non-communicable diseases in Hungary. The focus should be on preventing and controlling metabolic disturbances mainly contributing to the development of CVDs, type 2 diabetes and malignant diseases (14, 15).

The basic document for health promotion, the Ottawa Charter (16) emphasizes that health promotion actions have “to be facilitated in schools, homes, work places and community settings” because “health is created and lived by people within the settings of their everyday life.” A health promoting university (HPU) project was published by the WHO Regional Office for Europe in 1998 (17). In 2015 the Okanagan Charter (18) called on higher education institutions to incorporate health promotion values and principles into their mission and practice, however, information regarding how universities put these frameworks into actions is scarce. Recently a study by Suarez-Reyes et al. (19) analyzed how the HPU framework was implemented in 54 universities from 25 countries by using an online questionnaire. The action areas and items of work were defined and multi correspondence and cluster analysis was used to identify the types of universities based on the implementation of the HPU components. Their results demonstrated that universities implement the HPU framework for action very differently. In general, it can be stated that university/college health promotion programs predominantly target the university/college population (students and employees) as a whole with organized campaigns to promote healthy lifestyle among them (19). Authors who evaluated health promotion programs at college settings (20) clearly state that although the college setting offers some advantages for implementing health promotion programs, but they may also have unique challenges due to their large and diverse employee population. In addition, it is also concluded by them that “there is little research to show the effectiveness and unique challenges of college-based health promotion programs” (20).

## Context

### Semmelweis University's “Caring University” Initiative

The Semmelweis University is a leading institution in Hungary as well as in the Central European region within the area of medicine and health sciences. The University has six faculties (Medicine, Dentistry, Pharmaceutical Sciences, Health Sciences, Health and Public Administration, András Peto Faculty on Conductive Education), nearly 8,500 employees, and in addition to international teaching activities at undergraduate, graduate and postgraduate levels for around 11,000 students (one third of them from foreign countries), it is the largest provider of health care services in Hungary. The leadership of the Semmelweis University has realized the very severe and very complex public health challenges of Hungary and the advantages of workplace health promotion programs and decided to develop a new model program based on the HPU initiative, adapted to a health sciences university environment. The Semmelweis “Caring University” Model Program serves dual purpose. First, it is designed to improve the health of the university's staff and students using cutting-edge approaches of public health and preventive medicine. Second, it also serves as a pilot project for health promotion/disease prevention programs of medical and health colleges at occupational settings. In addition, its certain elements may serve as examples for the ongoing Hungarian primary health care reform.

The Semmelweis University's “Caring University” initiative to protect, promote health and prevent diseases among the employees of the University is based on a conceptually new approach. In addition to targeting the population of their employees as a whole, the concept of a more personalized prevention both at primary and secondary levels was introduced, which serves as basis to the development of its “Caring University” model program. This program targets all the employees with a sustainable and equitable, continuously operating preventive service provision. The participation in the Program is open for all University staff voluntarily, i.e., provided for everyone. The University leadership invites all its employees to take part in the Program by emphasizing the need of health promotion and disease prevention actions focusing on keeping people healthy. The staff members of the Departments of Public Health (as coordinator), Family Medicine, Behavioral Sciences, Physiotherapy, Dietetics and Nutritional Sciences, as well as that of the Health Promotion Center are actively involved in the recruitment of participants by winning the heads of all university departments to encourage the participation of their staff.

### Setting and Community Targeted

#### Center of Preventive Services: Goals and Structure

Based on the infrastructure and academic human resources of the Semmelweis University a multifunctional service center (Center of Preventive Services–CPS) is created which adopts the concept and model of reorientation of primary care into public health services.

The goal of the CPS is to provide integrated preventive services, including health promotion programs, health status assessment, lifestyle counseling and medical risk assessment. The blueprint of the CPS was originally developed and implemented in the framework of the Swiss Contribution Programme SH/8/1 titled “Public Health Focused Model Programme for Organizing Primary Care Services Backed by a Virtual Care Service Center” integrating the aforementioned services with traditional patients' care through general practitioners' (GPs') clusters (21, 22). The WHO Regional Director for Europe, Zsuzsanna Jakab praised the program in an Editorial of the European Journal of Public Health and expressed her hope that this work in Hungary would inspire many more such approaches elsewhere in the other 52 European Member States of WHO (23).

In the CPS model health professionals (medical specialists in preventive medicine and public health, non-medical public health professionals, physiotherapists, dieticians, health psychologists employed by the University as staff members of its Medical and Health Sciences faculties) competent to plan and implement various public health services collaborate with partner general practitioners and their practice nurses.

**Figure 1:**
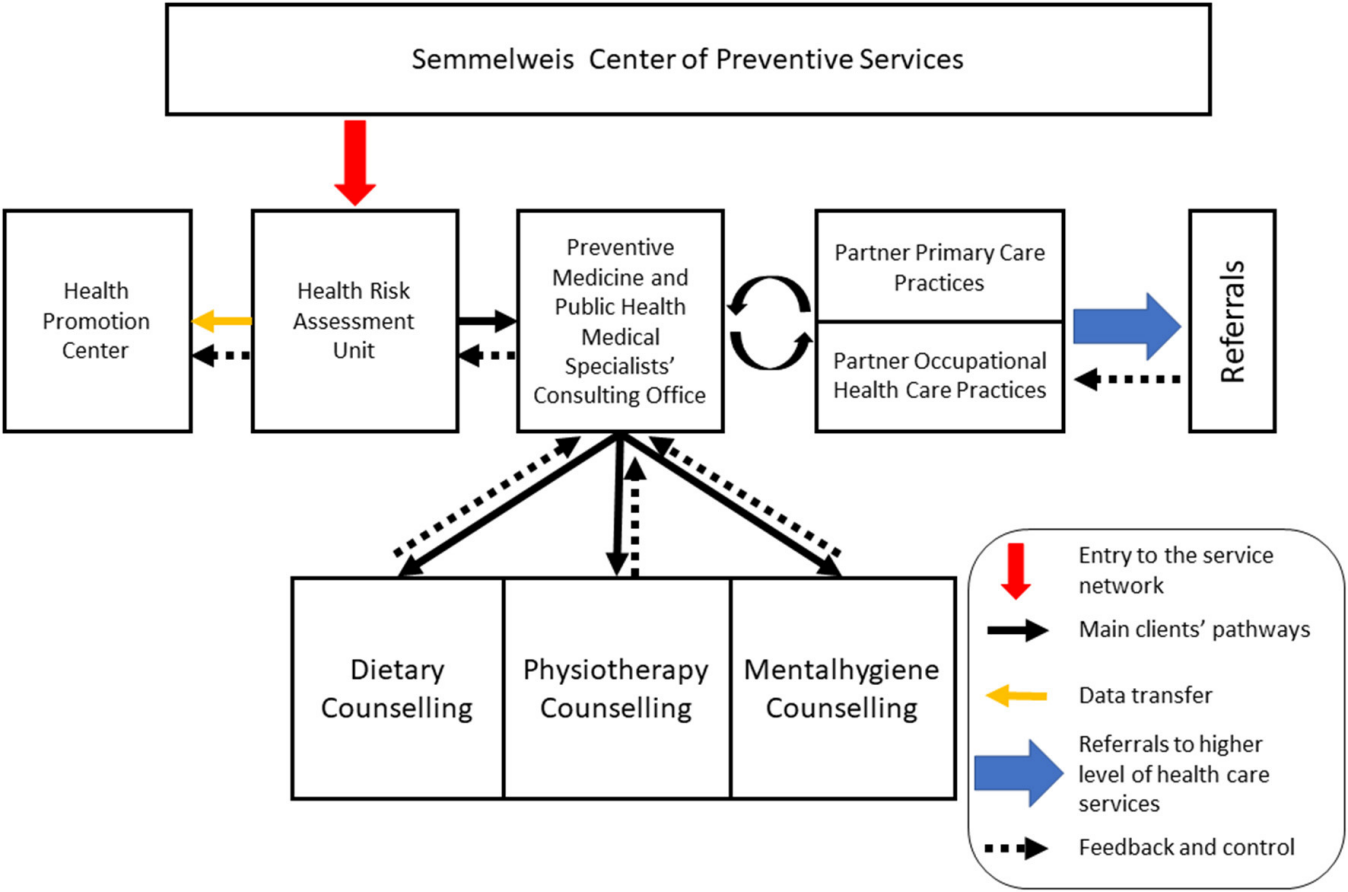
Structure of the Semmelweis Center of Preventive Services.

The CPS being developed at Semmelweis University extends the original blueprint and is composed of five main units which are in close collaboration with partner primary care practices and the occupation health care service of the University (Figure 1).

The costs of the operation of the Program are being covered from the University's central budget, while certain surveys built on it are financed from grants of the Ministry of Innovation and Technology. Medical services provided in the Program are covered by the National Health Insurance Fund with which the University as a health service provider has a contract. In addition the CPS acts also as a didactical infrastructure and the costs of operation are being covered partly from the resources for teaching.

## Key Programmatic Elements

The following services are to be provided by the units of CPS:

### Health Risk Assessment

This unit serves as the entry point into the network. Preliminary risk assessment will be carried out by using a questionnaire based on the European Health Interview Survey (EHIS) wave 2 which consists of four modules on (a) health status, (b) health care use, (c) health determinants, and (d) socio-economic variables. In these modules, the following topics are covered: (a) self-perceived health, chronic diseases known by the respondents, limitation in activities, and mental health, (b) use of different types of health care services, including hospitalizations, consultations, preventive (among them screening) services, and medications, and unmet needs for health care, and (c) smoking and alcohol consumption, physical activity, and dietary habits and additional background variables on demographics and socio-economic status such as sex, age, living conditions, education, income, and employment (24). In addition anthropometric (weight, height and waist circumference) and blood pressure (BP) measurements will be obtained for each participant by using the European Health Examination Survey protocol (25). After overnight fasting blood samples will be taken for measurement of key laboratory parameters, including total cholesterol, HDL-C, LDL-C, triglyceride, and glucose. Laboratory tests will be carried out in the Central Laboratory of the University. In addition to the calculation of the body mass index (BMI) the body composition will be determined. Individuals with health risks identified will be sent to the medical consulting office.

### Consultation With Medical Specialists in Preventive Medicine and Public Health–Medical Health Risk Assessment

After careful review of the health risk assessment record further risk assessment examinations (especially for clarifying CVD, diabetes and cancer risks) will be carried out and if it seems to be of additional diagnostic benefit after overnight fasting blood samples will be taken for laboratory tests to measure creatinine, uric acid, C-reactive protein, apolipoprotein A1, apolipoprotein B100 concentrations, alanine aminotransferase, aspartate aminotransferase, gamma-glutamyl-transferase, alkaline phosphatase activities, folic acid, Hemoglobin A1c, and insulin concentrations. Laboratory tests will be carried out in the Central Laboratory of the University.

### Dietary, Physiotherapy, and Mental Hygiene Counseling

If the medical risk assessment confirms that the health behavior and consequently the health status of the employee can be improved by life-style counseling he/she will be sent to the specific counseling unit(s) with detailed description of the problems identified. This service is available to clients by direct referral from medical specialists in preventive medicine and public health. In *dietary counseling* interventions are provided for overweight and obese persons, for persons at risk for cardiometabolic diseases and/or cancer, and special counseling for prediabetic/diabetic patients, as well as for persons with disturbances in lipid metabolism will also be given. *Physiotherapy counseling* will be provided for employees with locomotor problems among them for individuals with chronic low back pain, as well as for overweight or obese individuals. Special programs for clients with cardiorespiratory problems should also be considered. *Mental health promotion counseling* would be recommended for employees with signs of sleeping disorders, mood disorders, anxiety and stress-related disorders and substance abuse. Counseling can identify the need for further examinations by psychiatrist for schizophrenia, bipolar disorder or personality disorder. This service can be proposed for clients also for loss and bereavement management. Mental health promotion counseling also provides group-based services for stress-management and burnout prevention.

Combination of life-style counseling services (for example in case of body weight control programs), as well as group therapy (especially if it has advantages in comparison with individual therapy) for clients with the same problems will be considered and indicated by the medical specialist as a consequence of medical health risk assessment. The counseling methods will be tailored to the specific level of health literacy of the client.

### Health Promotion Center

The Health Promotion Center was founded in 2019, as part of the first phase of the implementation of the Caring University initiative. It operates as a central unit responsible for organizing coordinated and comprehensive health promoting programs in collaboration with the Department of Public Health mainly at community level for employees and students. Examples of the workplace health program components and strategies already implemented or being implemented include health education classes and university-wide health promotion programs open to students and staff, including fitness and recreation programs, programs that promote behavior change through gamification, programs that enhance resilience, interventions to prevent and reduce the negative effects of stress, as well as programs to reduce alcohol and other drug-related risk and harm on campus. A key mission is to highlight the role that healthy nutrition and physical activity play in supporting academic success and personal health. There are related programs in place to promote a healthy work environment through actions such as making healthy foods available and accessible through campus cafeterias. Within this framework the university also provides access to both university-owned and local fitness facilities and implement policies that promote healthy behaviors (e.g., limiting tobacco use on campus).

Partner primary care services and the occupational health care practice of the University will be involved in case of clients suspected to be diseased and/or need to be referred to higher levels of healthcare.

**Figure 2:**
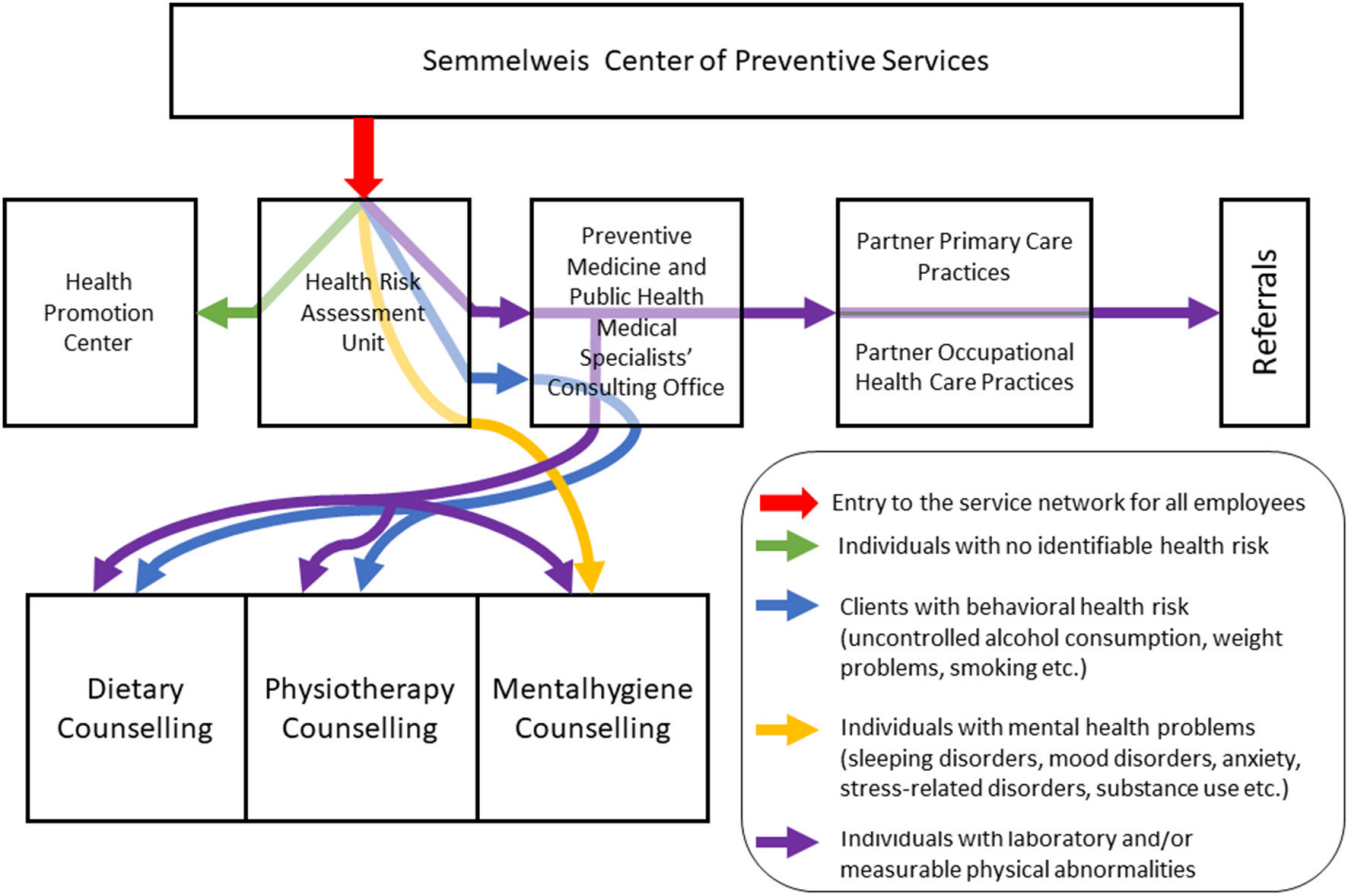
The employees' pathways in the Semmelweis Center of Preventive Services' network.

Regarding the employees' pathway the health status assessment unit represents the entry point into the CPS network (Figure 1). Individuals with no identifiable health risks will be instructed to participate in programs organized by the Health Promotion Center. Those with behavioral risks and/or laboratory or measurable physical abnormalities will be referred to the Preventive Medicine and Public Health Medical Specialists' Office. Based on the results of this medical risk assessment and further exploration of the risk status of the employees, they will either be sent to the relevant life-style counseling unit(s) of the CPS. Individuals with mental health problems will be oriented first to the mental hygiene unit where further specific examinations (for example specific tests to define depression or other mental disorders or sleep-wake problems) can be carried out. Those with suspected diseases will be oriented to the partner primary care practices for further diagnostic tests and examinations, defining medication or referrals to higher level of health care services (typically to the relevant specialists, but also to hospital care) as if it is necessary (Figure 2). In-person and virtual communications between CPS's units will be available at a regular basis.

The CPS operates in concerted actions of the relevant departments of university faculties (Table 1). The units of the CPS serve as practical training sites for medical students, residents in a clinical residency program in Public Health and Preventive Medicine, as well as for students in BSc in Public Health, BSc and MSc in Physiotherapy, and BSc and MSc in Nursing courses, as well for PhD students working on relevant topics.

**Table 1:** Faculties and departments of the Semmelweis University participating in the Caring University Model Program.

	**Participating Departments**
Faculty of Medicine*	**Department of Public Health (coordinator of CPS)**
	**Department of Family Medicine**
	**Institute of Behavioral Sciences**
Faculty of Health Sciences*	**Department of Dietetics and Nutritional Sciences**
	**Department of Physiotherapy**
	Department of Addictology
	Department of Nursing
	Department of Social Sciences
	Department of Public Health Sciences
Faculty of Health and Public Administration	Health Services Management Training Center
	Institute of Digital Health Sciences
	Institute of Mental Health
Faculty of Dentistry*	
Faculty of Pharmacology*	
Health Promotion Center	

The academic staffs of the departments indicated in bold are the main actors in the CPS. Faculties whose students participate in the teaching activities of the Caring University Model Program, including the health risk assessment, are labeled with “^*^”.

## Discussion

It has been well-established that health and well-being of the population, especially of working people, are crucial prerequisites for productivity and are of utmost importance for overall socioeconomic and sustainable development (26). Health promotion and disease prevention programs at occupational settings have a direct effect on the health of employees and by this way strongly contribute to the prosperity of institutions serving as their workplace (27, 28). Morbidity and mortality caused by non-communicable diseases are traditionally very unfavorable in Hungary which can be largely attributed to non-existent national public health program and primary care provision with no financial incentives to deliver preventive services (29). At an occupational setting an assessment to define employees' health and health risks followed by reflective interventions and/or adequate treatment (in collaboration with partner primary care providers) as well as monitoring the effects of these interventions is a good framework not only for improvement of health status of employees, but it has the potential to impact areas such as productivity and recruitment/retention ability of the employing institutions, reduction of absenteeism and reduction of health care costs at social level, as well.

The Semmelweis University as the leading training institution in the field of medical and health sciences of Hungary decided to create a public health model program for its employees at occupational settings. The core component of the program is a Preventive Service Center (CPS) including units with well-defined functions and services.

The Semmelweis Caring University Model Program and the operation of the CPS provide several benefits. First, they act as convincing evidence that the University recognizes the value of its employees and considers their health and well-being as a key issue. Second, they demonstrate that the University understood the link between the control of risks, the health and well-being of employees and the success of the organization itself. Third, they increase the reputation and attractiveness of the University, further strengthens the prestige of the “Semmelweis University” brand name. Fourth, they serve as models for other universities/colleges at national and international level. Recommendations can be specifically formulated for them, involving their staff in medical and health sciences trainings and provision of public health and health care services. Fifth, the units of the CPS serve as a training ground for teaching interdisciplinary team skills for students of the Faculties of Medicine, Pharmaceutical Sciences, Dentistry and Health Sciences. Sixth, the CPS also serves as a pilot project for the ongoing Hungarian primary health care reform.

In addition, the Semmelweis Caring University Model Program and the CPS will also benefit the research mission of Semmelweis University. The population of employees will be used as a cohort of a longitudinal epidemiological study, similar to the Whitehall Study II population (30, 31). The infrastructure of the Semmelweis Caring University Model Program and the CPS will serve as the basis to the development of the Semmelweis Study aiming to better understand the protective factors and barriers of healthy aging in Hungary. In this unique cohort study public health initiatives promoting healthy aging can be evaluated, facilitating the translation and dissemination of geroscience discoveries into sustainable, evidence-based public health programs and system-level strategies (32). The cohort can also be used as sampling frame to *ad hoc* surveys.

Monitoring and to define effectiveness of a workplace health promotion/disease prevention program is an essential requirement, although evaluation methods derived for and applied within the field of health promotion at different (especially occupational) settings have often fallen short of the ideal. In the most recent systematic review and meta-analysis carried out by Peñalvo et al. (33) could extract sufficient information to calculate pooled estimates for 20 different outcomes, of which 13 were found positively affected by workplace health promotion/disease prevention programmes, and could demonstrate beneficial effects on dietary factors, body anthropometrics, and cardiometabolic risk. These outcome indicators will be evaluated regularly every 5 years. Concerning the fact that in the Semmelweis Caring University Model Program the key programmatic elements are taken over from the “Public Health Focused Model Programme for Organizing Primary Care Services Backed by a Virtual Care Service Center” (21, 22), both process and outcome indicators on preventive service delivery are available to monitor the implementation and effectiveness of the Model Program proposed (29, 34). Process indicators (as attendance of the participants, program delivery in comparison to that which was intended, as well as an assessment of whether health-related interventions were implemented at the worksite, etc) will also be followed on a regular basis. A formative evaluation to see whether these indicators are in line with the assumptions will be carried out yearly.

## Author Contributions

ZU: conceptualization and writing–original draft. RÁ: conceptualization, writing–original draft, and visualization. AS: conceptualization, project administration, and writing–review and editing. GD, MM, GP, LK, and MK: writing–review and editing. PV: writing–review and editing and project administration. PT: conceptualization and writing–review and editing. BM: conceptualization, writing–review and editing, supervision, and funding acquisition. All authors contributed to the article and approved the submitted version.

## Funding

This work was supported by grants from the National Research, Development and Innovation Office (Nemzeti Szívlabor), the Hungarian Academy of Sciences (TK2016-78), and the European University for Well-Being (EUniWell) program (grant agreement number: 101004093/EUniWell/EAC-A02-2019/EAC-A02-2019-1). Project no. 135784 has also been implemented with the support provided from the National Research, Development and Innovation Fund of Hungary, financed under the K_20 funding scheme.

## Conflict of Interest

The authors declare that the research was conducted in the absence of any commercial or financial relationships that could be construed as a potential conflict of interest. The handling editor declared a past co-authorship with one of the authors RÁ.

## Publisher's Note

All claims expressed in this article are solely those of the authors and do not necessarily represent those of their affiliated organizations, or those of the publisher, the editors and the reviewers. Any product that may be evaluated in this article, or claim that may be made by its manufacturer, is not guaranteed or endorsed by the publisher.
